# Nasal Reconstruction

**Published:** 2013-02-11

**Authors:** Tom Reisler, Margaret H. Mysliwiec, Gregory L. Borah

**Affiliations:** ^a^Division of Plastic Surgery, Robert Wood Johnson Medical School, University of Medicine and Dentistry of New Jersey, New Brunswick, NJ, USA; ^b^Department of Medicine, University Hospital and New Jersey Medical School, Newark, NJ, USA

## DESCRIPTION

**Figure 1:**
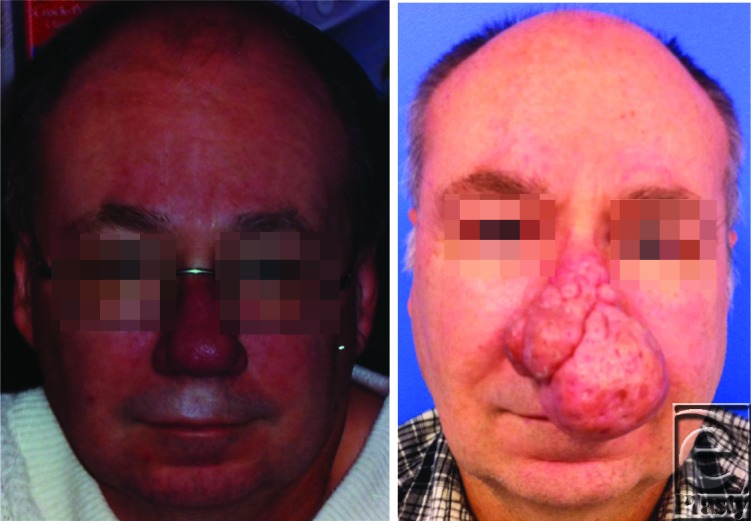
An old photograph before developing the nasal deformity (*left*); recent preoperative photograph (*right*).

A 55-year-old man comes to the office for consultation regarding a gradually enlarging lesion on his nose, and nasal airway obstruction. This has developed over several years. Physical examination shows enlargement of the nose, pitting and scarring of the skin, and several irregular and nodular growths (Fig 1).

## QUESTIONS

1. **What is the diagnosis?**
2. **What disease causes this condition?**
3. **What is the association of skin cancer with this skin condition?**
4. **What is the most appropriate management?**

## DISCUSSION

The patient has rhinophyma, or sebaceous hyperplasia of the nasal skin. Rhinophyma is believed to be the fourth stage of evolving rosacea. It is the proliferative phase that develops after acne rosacea. Patients have a predisposition to increased facial vascularity that can result in prerosacea or frequent facial flushing, vascular rosacea with erythrosis and telangiectasias, followed by inflammatory rosacea or acne rosacea, and ultimately rhinophyma.^1^^,^^2^

Patients typically present with nasal skin erythema and telangiectasias. In severe cases, the skin can have pits, fissures, and scarring. Sebum and bacteria can result in chronically infected skin and often an unpleasant odor. The nasal tip is preferentially enlarged, and as the nasal skin hypertrophies, the aesthetics units of the nose are distorted and obliterated.^3^ The skin lesions are not considered premalignant; however, a 3% to 10% incidence of occult basal cell carcinoma as been reported.^4^

Rhinophyma is treated either nonsurgically or surgically. Isotretinoin is indicated for acne rosacea and early rhinophyma.^2^ Dermabrasion alone can be used in mild to moderate cases of rhinophyma. However, any therapy involving ablation such as dermabrasion, cryotherapy, or laser therapy without tissue sampling is contraindicated. These 3 techniques are used as an adjunct to tangential excision of the affected skin to finely reshape the nose.

**Figure 2:**
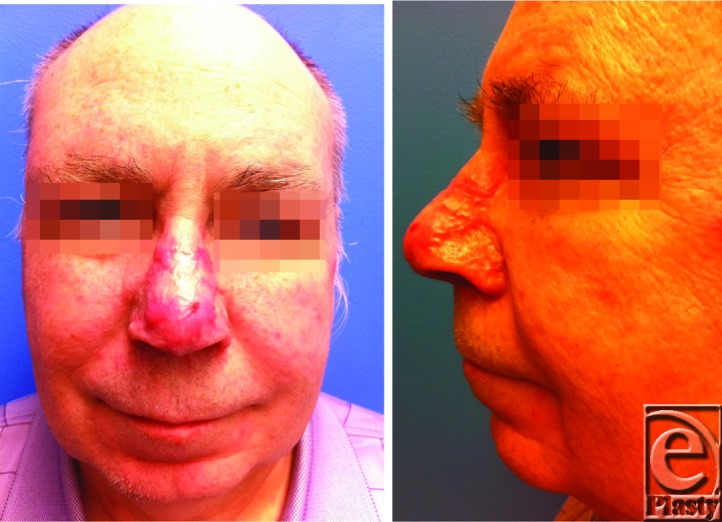
Several weeks postoperative photographs.

The most appropriate management for the patient described earlier is debulking via tangential excision by shaving of the affected skin, dermabrasion for final contouring, and cautery therapy for hemostasis. The wound heals by second intention due to the dermal appendages that are preserved following tangential excision, that is, reepithelialization ensures and completes in 2 to 4 weeks. Postoperative wound care includes application of petrolatum gauze dressing for up to 3 weeks (Fig 2).

In cases where isotretinoin is used, patients must wait 1 year after discontinuation of the drug before undergoing tangential excision, dermabrasion, or laser resurfacing because isotretinoin destroys sebaceous glands and would prevent reepithelialization.

Full-thickness excision with grafting is reserved for severe, recurrent cases of rhinophyma.
